# Non-Impacted Third Molars: Angels or Devils?

**DOI:** 10.3390/jcm12134455

**Published:** 2023-07-03

**Authors:** Rui-Xin Wu, Bei-Min Tian, Rui Gao, Fa-Ming Chen

**Affiliations:** State Key Laboratory of Military Stomatology and National Clinical Research Center for Oral Diseases, Department of Periodontology, School of Stomatology, Air Force Medical University, Xi’an 710032, China

Third molars, also known as wisdom teeth, are located in the most posterior of the tooth arch. They are the last teeth to erupt during one’s lifetime, which makes them the most likely to be impacted among all human teeth [1]. Third molars, especially impacted third molars, are associated with many oral pathologies such as pericoronitis, caries, alveolar bone resorption, root resorption, cysts and tumors. Therefore, it is a common practice to remove an impacted third molar when the tooth itself is diseased or severely threatens the health of the adjacent tooth and tissues. Due to the complexity of removing an impacted third molar, researchers have been trying to improve both preoperative assessment and postoperative treatment to minimize perioperative complications [2,3,4]. Despite the risk and difficulty of surgical extraction, if the patient’s local and general conditions permit, it is a clinical consensus that the (potentially) hazardous impacted third molars should be removed at an early age. However, whether to extract non-impacted third molars has always been a controversial topic.

Non-impacted third molars are those which have successfully erupted and vertically reached the normal occlusal plane. It’s estimated that 44.1% of the adult population have at least one non-impacted third molar [5], which could be kept as a reserve tooth for both prosthodontics (though third molars have become less necessary in prosthodontics with the advent of tooth implant techniques) and tissue regeneration. In traditional fixed bridge restoration, when the second molar (sometimes combined with the first molar) is missing, the third molar is often used as the distal abutment tooth, which solves the defect of the unstable single-end fixed bridge. In removable partial denture restoration, the third molar can also be used as the abutment tooth to place a partial retainer, bearing a portion of the occlusal force. Additionally, when the first or second molar is missing or irreparable, the third molar can be used as the donor tooth for auto-transplantation, which provides an alternative solution for dentition defects in clinical practice. Compared with impacted ones, non-impacted third molars are definitely the most suitable donor tooth, due to their easier extraction procedure and higher transplantation survival rate. Moreover, third molars have the potential to serve as stem cell banks [6]. Based on the accumulating achievements in dental-derived stem cell research, multiple stem cells have been isolated, including periodontal ligament stem cells, dental pulp stem cells, dental follicle stem cells, and so on. These stem cells, combined with well-designed biomaterials, play promising roles in regenerative medicine. Considering the various potential applications, it is no wonder that one might hesitate when deciding to abandon an asymptomatic non-impacted third molar. 

Despite the potential values of preserving non-impacted third molars, the accompanying adverse effects should not be ignored. Located in the backend of the dental arch, non-impacted third molars are inclined to receive insufficient oral hygiene measures and thus hold much plaque and debris, which would make the third molars subject to high risk of caries and even pulpitis. Besides the third molar itself, the adjacent second molar can also be involved. It has been found that, with the presence of non-impacted third molars, the incidence of adjacent second molar caries was higher than with the absence of non-impacted third molars (RR = 2.53) [7]. Worldwide, the prevalence of distal caries in second molars adjacent to non-impacted third molars is 7–10% [5,8]. Beyond increasing the decay risk of second molars, non-impacted third molars may also threaten the periodontal health of their neighbor teeth. According to a longitudinal study over 25 years, the second molar adjacent to an erupted third molar had a higher risk of developing a distal probing depth over 4 mm than the reference group in which the neighboring third molar was missing [7]. By reviewing the panoramic radiographs of 1958 outpatients, it was found that the prevalence of alveolar bone destruction exceeding 20% of the root length in second molars adjacent to non-impacted third molars was 40.4%. This was close to that in those adjacent to impacted third molars (41.5%) [5], suggesting that the non-impacted third molars might be as periodontally hazardous as the impacted ones. Cross-sectional studies and case follow-up studies further indicated the negative periodontal effects of non-impacted third molars to the adjacent second molars, showing that the risk of probing depth ≥ 5 mm (PD5+) in the second molars increased by 1.67~6.79 times when adjacent to non-impacted third molars, compared with those with neighboring third molar deletion [9,10,11]. A retrospective study which collected the clinical and radiographic data of 908 patients to analyze the loss reason of second molars discovered that the prevalence of the extracted second molars ascribed to periodontal diseases was significantly higher when the non-impacted third molars were present (42.3%) than when the third molars were missing or impacted [12]. However, a seemingly opposite conclusion was drawn by a retrospective cohort study, which stated that keeping a third molar was not related to the risk of losing its adjacent second molar in adult men [13]. It has been argued that previous studies considered second molar status alone, which would exaggerate the adverse effects of third molars, while ignoring the fact that the posterior teeth, especially the wisdom teeth, are inferior to anterior teeth in dental cleanliness and periodontal condition. We agree that the poor periodontal conditions of adjacent second molar areas are the consequence of combined factors, including the presence of non-impacted third molars and the hygienic dead corners caused by the posterior location. Studies have shown that, after removing the non-impacted third molars, the periodontal inflammation in the second molar area was relieved and indications were overall improved in clinical probing, immunology, and microbiology [11,14], which further supports the adverse periodontal effect of non-impacted third molars to their neighboring teeth. Although more evidence and prospective studies are needed, we boldly postulate that preventive extraction of non-impacted third molars would reduce the risk of irreversible damage to adjacent second molars.

The debate on how to manage non-impacted third molars still goes on, and the recommendations can vary from organization to organization, from dentist to dentist, based on the different standpoints and professional backgrounds. Clinically, we periodontists have definitely noticed the periodontal threat to the adjacent teeth brought about by non-impacted third molars. The periodontal damage caused by non-impacted third molars is related to many factors, such as age, oral regions, and even interdental alveolar bone width [9,10,15]. Consequently, we suggest that clinicians should pay attention to the potential harm caused by non-impacted third molars, closely monitor the condition of the adjacent second molars, and adopt an individualized approach to each patient after multidisciplinary evaluation.
